# The absolute CBF response to activation is preserved during elevated perfusion: Implications for neurovascular coupling measures

**DOI:** 10.1016/j.neuroimage.2015.10.023

**Published:** 2016-01-15

**Authors:** Joseph R. Whittaker, Ian D. Driver, Molly G. Bright, Kevin Murphy

**Affiliations:** a Cardiff University Brain Research Imaging Centre (CUBRIC), School of Psychology, Cardiff University, CF10 3AT Cardiff, UK; b Sir Peter Mansfield Imaging Centre, Clinical Neurology, School of Medicine, University of Nottingham, Nottingham, UK

**Keywords:** Functional MRI, Cerebral blood flow (CBF), Cerebral metabolic rate of oxygen consumption (CMRO_2_), Blood flow-oxygen metabolism coupling, Calibrated BOLD, Arterial spin labelling (ASL)

## Abstract

Functional magnetic resonance imaging (fMRI) techniques in which the blood oxygenation level dependent (BOLD) and cerebral blood flow (CBF) response to a neural stimulus are measured, can be used to estimate the fractional increase in the cerebral metabolic rate of oxygen consumption (CMRO_2_) that accompanies evoked neural activity. A measure of neurovascular coupling is obtained from the ratio of fractional CBF and CMRO_2_ responses, defined as *n*, with the implicit assumption that relative rather than absolute changes in CBF and CMRO_2_ adequately characterise the flow-metabolism response to neural activity. The coupling parameter *n* is important in terms of its effect on the BOLD response, and as potential insight into the flow-metabolism relationship in both normal and pathological brain function. In 10 healthy human subjects, BOLD and CBF responses were measured to test the effect of baseline perfusion (modulated by a hypercapnia challenge) on the coupling parameter *n* during graded visual stimulation. A dual-echo pulsed arterial spin labelling (PASL) sequence provided absolute quantification of CBF in baseline and active states as well as relative BOLD signal changes, which were used to estimate CMRO_2_ responses to the graded visual stimulus. The absolute CBF response to the visual stimuli were constant across different baseline CBF levels, meaning the fractional CBF responses were reduced at the hyperperfused baseline state. For the graded visual stimuli, values of *n* were significantly reduced during hypercapnia induced hyperperfusion. Assuming the evoked neural responses to the visual stimuli are the same for both baseline CBF states, this result has implications for fMRI studies that aim to measure neurovascular coupling using relative changes in CBF. The coupling parameter *n* is sensitive to baseline CBF, which would confound its interpretation in fMRI studies where there may be significant differences in baseline perfusion between groups. The absolute change in CBF, as opposed to the change relative to baseline, may more closely match the underlying increase in neural activity in response to a stimulus.

## Introduction

Functional magnetic resonance imaging (fMRI) based on the blood oxygenation level dependent (BOLD) effect is a useful tool for spatially mapping stimulus induced neural activity in the human brain. BOLD fMRI depends on the paramagnetic properties of deoxyhaemaglobin, which alters the susceptibility of blood and makes a T_2_^⁎^-weighted MR signal sensitive to blood oxygenation (Buxton, 2013). The magnitude of the BOLD signal is dictated by neurovascular coupling, the intrinsic relationship between cerebral blood flow (CBF) and cerebral metabolic rate of oxygen consumption (CMRO_2_), which ensures that the metabolic demands of brain function are satisfied. The fundamental physiological phenomenon producing the BOLD response is a divergence in CBF and CMRO_2_ changes, first observed in PET (Fox and Raichle, 1986), which results in a paradoxical increase in blood oxygenation downstream from the site of increased neural activity and causing an increase in the measured local MR signal. The haemodynamic nature of the BOLD signal permits only a qualitative estimate of neural activity, which makes interpretation and group level comparisons difficult. The non-specific nature of signal changes is particularly problematic for studies of patient populations, where pathological or atypical cerebral physiology may confound interpretation of the BOLD signal as a surrogate measure of evoked neural activity.

Arterial spin labelling (ASL) is a viable alternative to BOLD fMRI as a technique in which CBF is directly measured and quantified in absolute physiological units. The advantage of ASL is that a direct measurement of perfusion to the capillary bed provides better localisation of the site of neural activity, less intra- and inter-subject variability, and less sensitivity to baseline signal drifts, allowing low frequency experimental designs to be utilised (Detre et al., 2009). Combining simultaneously acquired measurements of BOLD and CBF responses to yield an estimate of CMRO_2_ is the objective of calibrated BOLD methods (Blockley et al., 2013, Hoge, 2012). It is generally assumed that CMRO_2_ is the parameter most closely related to neural activity (Buxton, 2013). The ATP production required for maintenance of sodium and calcium gradients during neural activity is the dominant source of energy expenditure in the human brain, and proceeds mostly by aerobic metabolism (Attwell and Laughlin, 2001). Calibrated BOLD methods are therefore promising in that they provide a more direct measure of neural activity in the form of CMRO_2_, as well as characterising the degree of neurovascular coupling, thus delivering a more complete assessment of brain function.

The ratio of fractional CBF and CMRO_2_ responses is defined in PET and MRI literature as *n*. The calibrated BOLD framework can be used to estimate the neurovascular coupling parameter *n*, with evidence from several studies suggesting that *n* may be modulated by a myriad of factors, such as age (Schmithorst et al., 2015), brain region (Ances et al., 2008, Chiarelli et al., 2007), attention (Moradi et al., 2012), adaptation (Moradi and Buxton, 2013), and stimulus intensity (Liang et al., 2013, Lin et al., 2008, Vafaee and Gjedde, 2000). These reports imply that *n* may confound interpretation of BOLD data if not accounted for, but may also be a potentially interesting facet of brain function in itself (Buxton et al., 2014). An incomplete understanding of neurovascular coupling mechanisms makes it unclear to what degree the variability in *n* that has been reported represents a fundamental feature of brain physiology.

Global baseline CBF is a potential confound for fMRI as it can be perturbed in certain patient populations, but also in healthy subjects by commonly used substances (e.g. caffeine or nicotine), or changes in endogenous chemical concentrations (e.g. adrenalin or oestrogen) (S. G. Kim and Ogawa, 2012). Thus an effect of baseline CBF on the magnitude or dynamics of BOLD and CBF responses could lead to false inferences in studies using subject groups with significantly different baselines. There have been numerous studies examining this issue (Cohen et al., 2002, Corfield et al., 2001, Kastrup et al., 1999, Liau et al., 2008, Posse et al., 2001, Shimosegawa et al., 1995, Uludag et al., 2004), but no clear consensus has been reached on how baseline CBF impacts on steady state BOLD and CBF responses in the healthy human brain. Although CBF/CMRO_2_ coupling as defined by *n* is a widely accepted metric in PET and MRI literature, the impact of possible baseline effects on *n* remains relatively unaddressed.

In the work presented here we use a graded visual stimulus to characterise the variability of *n*, both within and between different levels of baseline perfusion, which we perturb using a hypercapnia challenge. Carbon dioxide (CO_2_), a potent vasodilator routinely used to robustly modulate CBF, is used in this study to create a state of global hyperperfusion in healthy human subjects. One of the advantages of using ASL is the ability to quantify CBF in active and baseline states, which allows us to fully characterise the brain's hyperaemic response to a stimulus in absolute terms. In measuring the CBF response to the same stimulus at different baseline levels, we can then determine whether relative or absolute CBF best reflects the underlying evoked neural activity. If fractional CBF changes are dependent on baseline then this affects the coupling parameter *n*, and raises the question; how informative is *n* as a measure of neurovascular coupling?

## Methods

### Theory

In this section we review the calibrated BOLD framework. Table 1 defines the variables referred to throughout.

Stimulus evoked fractional changes in CMRO_2_ (%ΔCMRO_2_) can be estimated within the calibrated BOLD framework, based on the model introduced by Davis et al. (1998), which relates CMRO_2_ and CBF in active and baseline states to %ΔBOLD. The model for the extravascular BOLD signal change following neuronal activation is given as:

(1) $$\frac{\Delta S}{S_{0}}=M\left[1-{\left(1+\frac{\Delta CBF}{CBF_{0}}\right)}^{\alpha -\beta }{\left(1+\frac{\Delta CMRO_{2}}{CMRO_{2|0}}\right)}^{\beta }\right]\text{.}$$

*M* is a scaling parameter that represents the maximum possible BOLD signal change that would be achieved by complete elimination of all deoxyhaemaglobin. It comprises a number of factors determined by the baseline physiological state, as well the dependence of ΔR^⁎^_2_ on echo time, and thus is specific to a subject and region during a particular scanning session. In the original derivation, the parameter α models the relationship between cerebral blood volume (CBV) and CBF based on animal studies (Grubb et al., 1974), and is given a value of 0.38. The parameter β is the power law relationship between deoxyhaemoglobin and ΔR^⁎^_2_, which is dependent on the diameter of blood vessels, and was originally determined to be 1.5 for a field strength of 1.5 T based on Monte Carlo simulations (Davis et al., 1998). Recent simulations using a multi-compartment model for the BOLD signal, found that optimised values for α and β of 0.14 and 0.9 respectively for 3 T improved the accuracy of the Davis model in predicting steady-state BOLD signal changes (Griffeth and Buxton, 2011). These new values of α = 0.14 and β = 0.9 were adopted for this study.

With simultaneous measurements of %ΔBOLD and %ΔCBF there are still two unknowns in the Davis model, %ΔCMRO_2_ and M. The traditional approach to calibrate the BOLD signal is to perform a separate hypercapnia experiment, whereby CO_2_ causes global vasodilation that results in BOLD and CBF signal changes (%ΔBOLD_HC_ and %ΔCBF_HC_) without a change in CMRO_2_. By assuming that hypercapnia is iso-metabolic (i.e %ΔCMRO_2_ = 0), the CMRO_2_ term is equal to 1, and therefore M can be calculated. Subsequent %ΔBOLD and %ΔCBF for a neuronal stimulus can be calibrated using the hypercapnia derived M to obtain %ΔCMRO_2_.

### Subjects

Ten healthy adults (7 males, mean age 30.1 (range 25–37)) participated in this study, which consisted of one scanning session of two functional runs. One subject completed only one run due to scanner technical problems, and one subject completed the second run in a separate session approximately 3 h after the first session. All participants gave written, informed consent. The study was approved by the School of Psychology, Cardiff University Ethics Committee.

### Paradigm

**Fig. 1:**
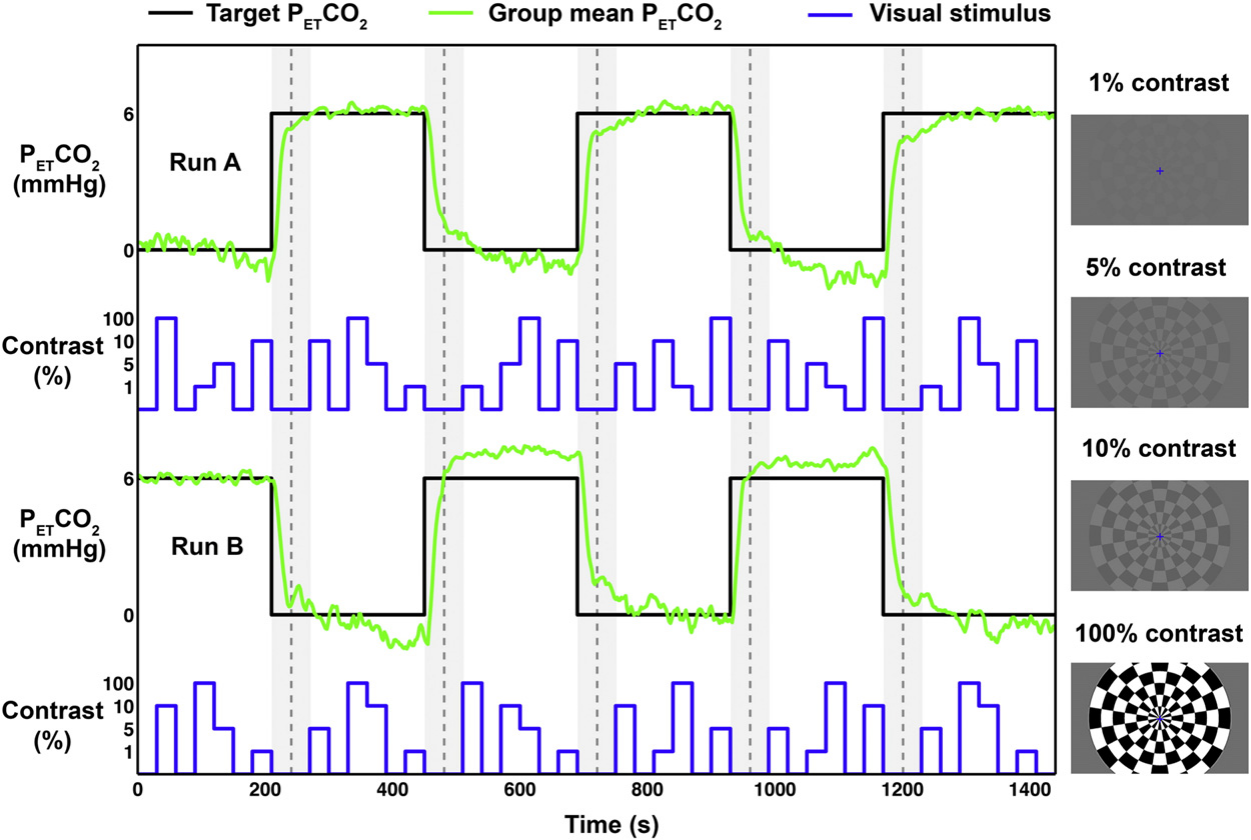
Schematic of experimental design of the two functional runs. Block design of hypercapnia stimulus for each run (black) along with group average P_ET_CO_2_ values (green), and block design of graded visual stimulus (blue). Dashed grey lines delineate different baseline periods, and light grey shaded area indicates minute long transition periods between baseline states. Blue trace represents example contrast levels, which were presented in a pseudorandom order for 30 second periods, with each contrast being presented for each baseline block.

The full experimental paradigm, which consisted of a visual activation task and hypercapnia challenge, is illustrated in Fig. 1. Two 24-minute functional runs each consisted of a block design visual stimulus, and a block design hypercapnia challenge. The visual stimuli were projected onto a screen inside the scanner bore, which was viewed by subjects via a mirror attached to the head coil. The stimulus was a radial checkerboard reversing at 8 Hz that was presented for 30 s at four different isoluminant contrast levels, 1%, 5%, 10%, and 100%. These levels were chosen to maximise the dynamic range of the BOLD response, which is known to be a nonlinear function of contrast best fit by a power law relationship (Olman et al., 2004). During 30 second rest periods a blue fixation cross was presented at the centre of an isoluminant grey background.

The 24-minute functional run was split into 6 periods, each lasting 4 min. During each period, visual stimuli (V) and rest periods (R) were presented in the following order R–V_1_–R–V_2_–V_3_–R–V_4_–R, each lasting 30 s. Each V_i_ represents one of the 4 contrasts (1%, 5%, 10% and 100%), which were presented in a pseudorandom order across the 4 minute periods. At the same time, three blocks of normocapnia (NC) were interleaved with three blocks of hypercapnia (HC). The switches between NC and HC occurred directly after the last visual contrast stimulus (see V_4_ above) was presented in each period (therefore, switches occurred at 3.5, 7,5, 11.5, 15.5 and 19.5 min). In this way, there was a 1 minute period between the last visual stimulus at NC and first visual stimulus at HC (and vice versa). The minute long transition period (i.e. the last 30 s rest of one period and the first 30 s rest of the next period) was discarded during response calculations so that only steady-state gas levels were considered. Thus, across the entire 24-minute functional run, each contrast was presented six times, 3 during NC and 3 during HC. The pattern of NC and HC periods was reversed between runs for each subject, and balanced across subjects such that half of them began the first functional run with a block of HC.

### Respiratory challenge

A tight fitting mask was used to manually deliver gas through a system of flow metres. Manual control of flow of medical air and 5% CO_2_ was directed through separate humidifiers and allowed to mix in a length of tubing before reaching the mask. A sampling line connected to the facemask was used to monitor end-tidal CO_2_ concentrations.

A minimum gas flow rate of 30 L/min was maintained at all times. For NC periods, only medical air was delivered. During HC periods the flow of 5% CO_2_ was manually increased, with a corresponding decrease in flow of medical air to maintain 30 L/min total flow rate. The flow of CO_2_ was increased with the aim of raising subjects' partial pressure of end-tidal CO2 (P_ET_CO_2_) by + 6 mm Hg from a predetermined baseline level. Subject P_ET_CO_2_ was constantly monitored during HC periods, and small adjustments to flow rates were made as necessary to try to achieve a well-defined hypercapnia block design.

### Imaging protocol

Functional runs were acquired on a 3 T GE HDx scanner using an eight-channel receiver head coil. A pulsed arterial spin labelling (PASL) sequence (PICORE QUIPSS II) (Wong et al., 1998) (TI_1_/TI_2_ = 700/1500 ms, 20 cm tag width, 1 cm gap between tag and most proximal slice) with a dual-echo, gradient-echo spiral readout (TR/TE_1_/TE_2_ = 2200/3/29 ms, 64 × 64 × 8 matrix (3 × 3 × 7 mm^3^, 1 mm inter-slice gap), 655 volumes) was used to acquire both quantitative perfusion measurements and BOLD weighted images. For perfusion quantification a single echo equilibrium magnetization scan was acquired, with the same parameters as the functional run, minus the ASL preparation. Additionally, a minimum contrast scan (TE/TR = 11/2000 ms) was acquired to correct for field inhomogeneity.

The use of a global vasodilatory stimulus could theoretically lead to a systematic bias in quantification of CBF. As accurate quantification is paramount in this study, ASL parameters were carefully chosen to limit this possibility. The hypercapnia stimulus causes global vasodilation in the brain, and so decreases the time it takes (after the inversion pulse) for tagged blood to arrive at the imaging plane (δt). The QUIPSS II saturation pulse creates a bolus of known temporal width so that estimates of CBF are insensitive to δt, provided that TI_1_ < natural temporal width of the bolus, and TI_2_ > δt + TI_1_ (Wong et al., 1998).

A large tag width of 20 cm was chosen to ensure that the first condition was met for physiologically plausible range of blood velocities, even during hypercapnia. A relatively long TI_2_ of 1500 ms for the most proximal slice ensures that all of the tagged blood is delivered during normocapnia (when blood velocity is expected to be lower), so that no bias is introduced.

### Analysis

All data were analysed in AFNI (Cox, 1996). The first four images (8.8 s) of the functional scans were excluded to allow the MRI signal to reach a steady state. Images from both echoes were registered to the first images of the first functional run to correct for motion. A scaling factor (κ) that rendered the difference image in quantitative units of perfusion (mL 100 g^− 1^ min^− 1^) was applied to the raw un-subtracted data from both echoes, and is defined as $\kappa =M_{0B}\left(TI_{1}\right)e^{-TI_{2}/T_{1B}}e^{-TE_{1}/T_{2B}^{*}}$ (Liau et al., 2008). M_0B_ is the equilibrium magnetisation of blood (estimated from M_0_ of CSF), TI_1_ is the bolus width of the tag, T_1B_ is the longitudinal relaxation time of blood (assumed 1700 ms), T^⁎^_2B_ is the transverse relaxation time of blood (assumed 436 ms), and TE_1_ is the time of first echo. TI_2_ is the inversion time, which was adjusted on a slice-by-slice basis to account for increased T_1_ relaxation at more distal slices acquired at a later time.

For each echo and each run, a statistical analysis of the un-subtracted data was performed using a general linear model (GLM) approach (Mumford et al., 2006). For each of the 4 contrast levels, the block design was convolved with a HRF to create a visual stimulus regressor, with equal and unit amplitude for all contrasts, i.e. stimulus regressors encoded the size of each contrast response relative to baseline. Contrasts were considered separately for NC and HC, so there were two sets of regressors for each contrast level (8 visual regressors in total). For each functional run, P_ET_CO_2_ values were convolved with a HRF to make a regressor that modelled the response to the hypercapnia challenge. A vector of alternating − 1 s and 1 s was used to model the ASL tag/control modulation, and was multiplied by the other regressors to model changes in the ASL difference signal. Thus, BOLD and perfusion NC baselines, and responses to the visual stimulus and hypercapnia challenge are explicitly modelled. Also included were terms to model signal drifts (up to 3rd order Legendre polynomials), physiological noise (RETROICOR) (Glover et al., 2000, Restom et al., 2006), autocorrelation and Gaussian noise.

### ROI definition

To avoid a possible bias introduced by large draining veins, a region of interest (ROI) was defined using CBF activations alone (i.e. first echo data) (Leontiev et al., 2007). For each subject, ROIs were selected within an occipital lobe mask and were limited to grey matter voxels by choosing only those with significant tag/control modulation (p < 0.001, uncorrected), and a significant CO_2_ response (p < 0.001, uncorrected) in the first echo data. The ASL signal generally has poorer SNR in white matter, due to increased transit times and the fact that it is intrinsically less vascularised than grey matter (van Gelderen et al., 2008). This combined with the significantly higher CO_2_ reactivity in grey matter (Thomas et al., 2014), heavily weights our mask threshold criteria to grey matter voxels.

A t-contrast to test the linear combination of visual regressor model parameters was used to identify voxels that respond to all visual contrasts (i.e. a linear summation of all visual regression coefficients for both baseline conditions), using a threshold of p = 0.05 and a minimum cluster size determined by AFNI's *3dClustSim* function to control for multiple comparisons. A limitation is the need to average data over an ROI, most likely including multiple cell types that may be more or less sensitive to different luminance contrast levels. Thus, as the responses from all visual contrasts are treated equally in the t-contrast, the ROI is as unbiased to an individual contrast response as possible.

### Calculation of BOLD and CBF responses

The modelled physiological noise and BOLD effect were regressed out of the first echo (e1) data, and then surround subtraction was performed (SS) to create a quantitative CBF dataset (SS_e1_). The mean SS_e1_ signal within the ROI mask was then calculated. The modelled physiological noise and CBF effect were regressed out of the first and second echo (e1, e2) data, and then surround addition (SA) was performed to create two BOLD weighted datasets (SA_e1_, SA_e2_). Interpretation of BOLD signal changes is confounded by low frequency noise, which is particularly problematic for these data, as we are trying to estimate the signal change from a varying baseline that is modulated by slow changes in inspired CO_2_. To minimise this source of error we used the ROI mean of the first and second echo surround averaged data (SA_e1_, SA_e2_) to calculate R^⁎^_2_ with the equation *R*_2_^⁎^(*t*) = ln(*SA*_*e*1_(*t*)/*SA*_*e*2_(*t*))/Δ*TE*, where ΔTE is the difference between the first and second echoes.

**Fig. 2:**
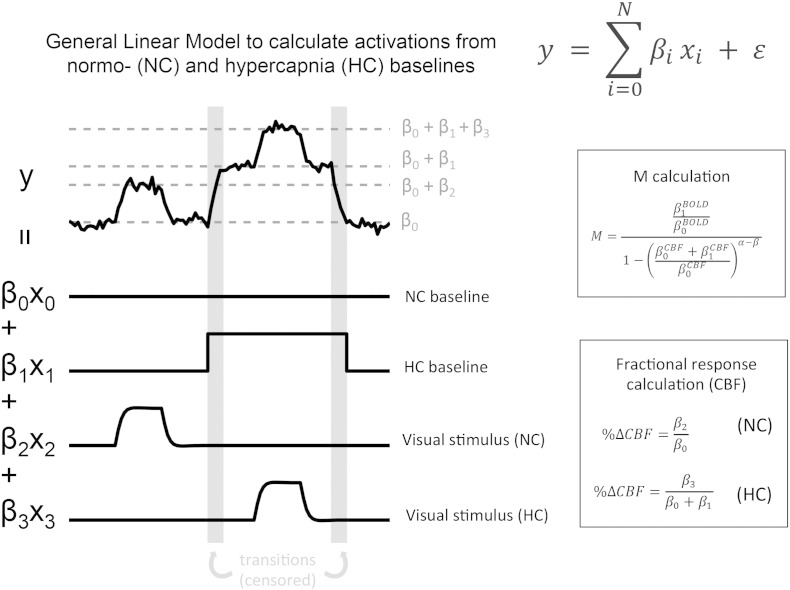
Schematic showing how fractional signal changes can be calculated from two different baselines (NC and HC) within a GLM framework. Different regression coefficients represent different baselines, and signal changes from respective baselines. The effect of hypercapnia can also be considered separately from the visual effects allowing M be calculated. This same model applies for average CBF, BOLD or R2* signals. N.B. This is for illustration only and does not reflect real data or the design of the experiment.

Estimates of R^⁎^_2_ and CBF signal changes to each of the four visual stimuli were obtained using a general linear model (GLM) of the mean CBF and R^⁎^_2_ time series respectively, with regressors made from the same visual contrast timings convolved with a HRF that were used for the voxel wide GLM. Modelling CBF and R^⁎^_2_ (or BOLD) signals as linear summations of different terms is ideal, as it can include different baseline levels, as illustrated in Fig. 2. The model included constant terms for the NC and HC baselines and the 1-minute transition periods were censored to ensure baseline estimations consisted only of steady states. The CBF model coefficients were in physiological units of mL 100 g^− 1^ min^− 1^ due to the scaling factor (κ) applied during pre-processing, and represented absolute CBF changes to stimulation (ΔCBF). Division of these absolute changes by their respective baselines yielded fractional CBF changes (%ΔCBF). The R^⁎^_2_ model coefficients were in units of change in R^⁎^_2_ from baseline (ΔR^⁎^_2_), and fractional BOLD changes (%ΔBOLD) were estimated using the equation $\%\Delta \mathrm{BOLD}=e^{-TE_{2}\Delta R_{2}^{*}}-1$, where TE_2_ is the time of the second echo.

### Estimation of CBF/CMRO_2_ coupling

ASL data typically suffer from poor SNR, which is problematic for use with the Davis model where error propagation is an issue. Therefore, estimations of visual stimulus evoked %ΔCMRO_2_ during the NC period were acquired in two ways, outlined below.

#### Full calibration approach

The traditional way uses %ΔBOLD_HC_ and %ΔCBF_HC_ to the HC challenge to calculate individual subject M values with the Davis model, which are then substituted back into the model to estimate %ΔCMRO_2_. In the same way that BOLD and CBF responses to the visual stimuli were calculated (see above), hypercapnia induced signal changes, that are independent of task induced responses, can be estimated from the GLM (see Fig. 2). We refer to this as the *full calibration* approach. Individual subject M values were estimated for each separate functional run, and then averaged for subsequent calculations as they are expected to be approximately equal for each run. Estimation of visual stimulus %ΔCMRO_2_ for each contrast level was performed separately for each run, using the run averaged values of M.

#### Ratio method

One of the primary aims of this study was to investigate whether changes in *n* occur with changes in stimulus intensity, and whether that is dependent on CBF_0_. As %ΔBOLD and %Δ CBF for each contrast level are from the same baseline level, *M* remains constant within NC and HC conditions respectively. Thus when we compare response between different contrast levels within condition, the *M* terms cancels, and so without estimating it we can infer changes in *n*. This was the approach used by Liang and colleagues (Liang et al., 2013). We refer to this as the *ratio method*. In this approach, %ΔCMRO_2_ for a given contrast level is calculated from the %ΔCMRO_2_ in a reference contrast (100%), and requires an assumed value of *n* for the 100% contrast.

Estimating visual stimulus %ΔCMRO_2_ for each contrast level in the HC condition is complicated by the fact that M is different from the NC conditions due to altered CBF_0_, and consequently venous cerebral blood volume (CBV_v_) and deoxyhaemaglobin concentration [dHb]. For this reason neither the *full calibration* nor the *ratio method* were used to estimate %ΔCMRO_2_ in the HC condition. Instead, we assume that the absolute change in CMRO_2_ (ΔCMRO_2_) to the individual visual contrasts is equal during the NC and HC conditions, and thus %ΔCMRO_2_ are also equal if the assumption of iso-metabolism during hypercapnia holds.

## Results

### BOLD and CBF activations

All subjects exhibited robust focal activation due to the visual stimulus and global signal changes in response to the HC challenge. The mean (± SD) hypercapnic change in P_ET_CO_2_ across all subjects and functional runs was 6.62 ± 0.85 mm Hg, which increased the ROI CBF from a mean (± SD) of 52.1 ± 8.60 to 78.5 ± 9.00 mL 100 g^− 1^ min^− 1^, and resulted in a mean (± SD) ROI BOLD signal change of 1.96 ± 0.71%.

**Fig. 3:**
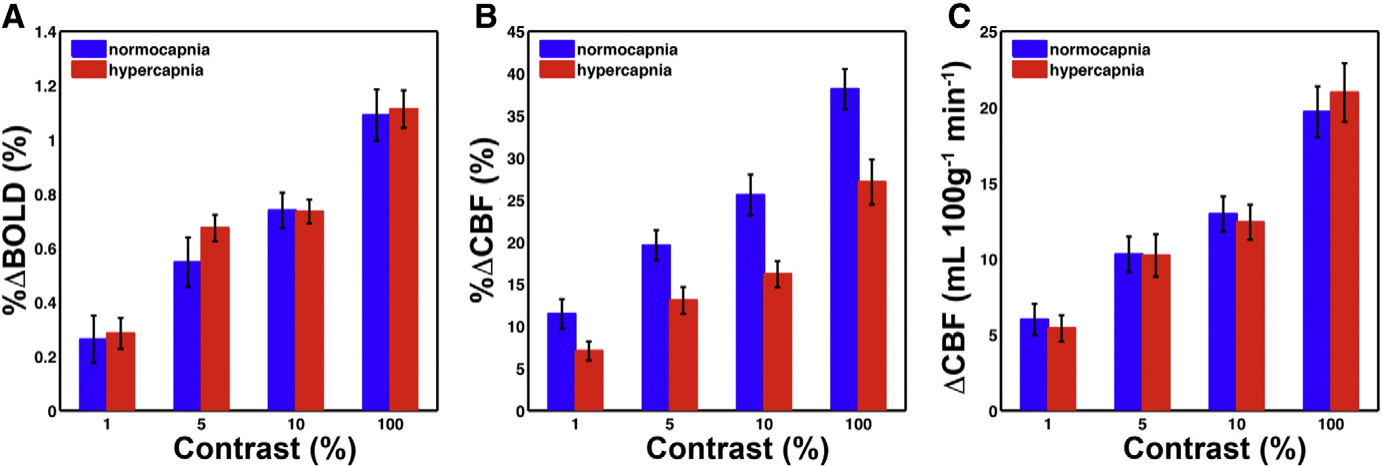
Group mean responses with standard error of the mean (SEM) error bars. A) Mean %ΔBOLD (± SEM) for each contrast level. B) Mean %ΔCBF (± SEM) for each contrast level. C) Mean ΔCBF (± SEM) for each contrast level.

Measured visual stimulus %ΔBOLD, %ΔCBF, and ΔCBF are given in Table 2, and shown in Fig. 3. One-way repeated measure ANOVA models with baseline condition and contrast as factors were constructed to look at their main effects on response magnitudes, as well as their interaction effect. In the presence of a main effect of contrast, a significant interaction effect between contrast and baseline would indicate a difference in the graded response to contrast across the different baseline levels. As expected %ΔBOLD, %ΔCBF, and ΔCBF all showed a graded response to contrast (p < 0.001), but baseline condition only had an effect on %ΔCBF, which had significantly smaller values in the HC condition (p < 0.001). There were no significant interaction effects, meaning %ΔBOLD, %ΔCBF, and ΔCBF all showed the same graded response to the different contrast levels in both baseline conditions, although offset by a set amount in the HC condition for %ΔCBF. Although small, %ΔBOLD for the 1% contrast were significantly different from zero (p < 0.05), with only two subjects showing negative responses.

### Estimates of %ΔCMRO_2_ and n

**Fig. 4:**
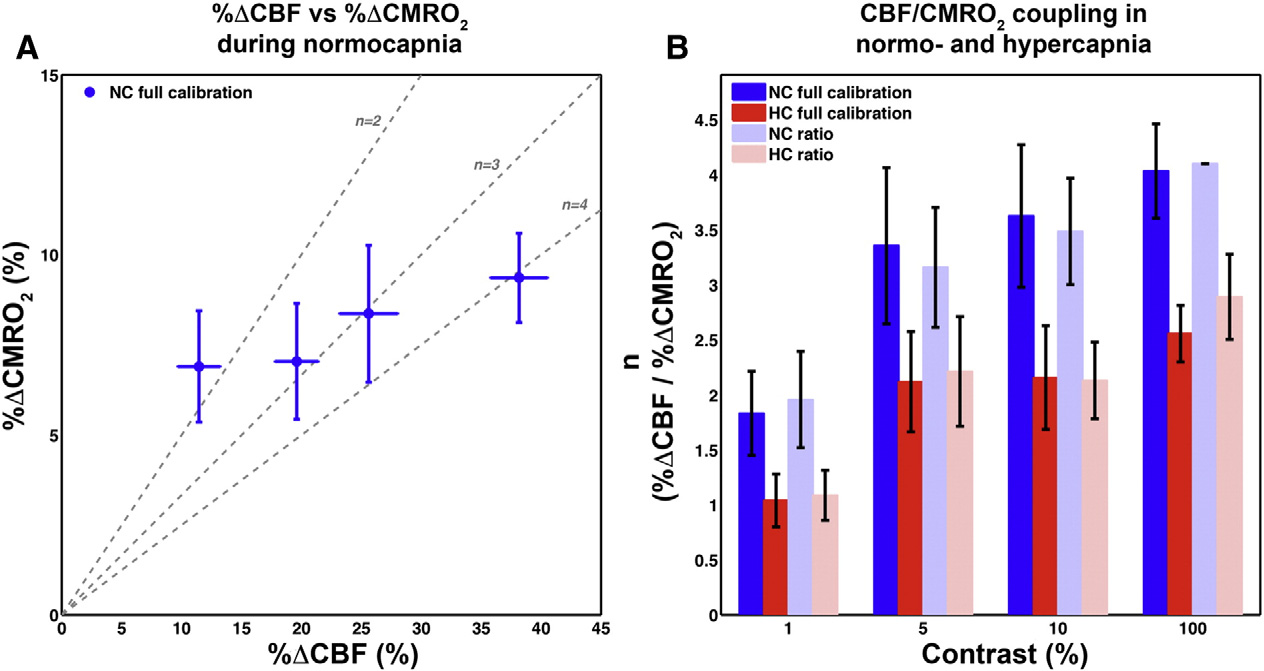
A) Mean *full calibration* %ΔCMRO_2_ estimates (± SEM) vs. measured %ΔCBF in NC condition. Dotted grey lines show iso-*n* trajectories in CBF/CMRO2 coupling space. B) NC and HC *full calibration* and *ratio* method n values for different contrast levels.

The group average M value was 7.3 ± 1.4% (mean ± SD), which is in good agreement with the literature for the field strength and echo time (Hare and Bulte, 2015), and CBF derived ROI (Leontiev et al., 2007). Estimates of %ΔCMRO_2_ using the *full calibration* method were averaged across the two runs for subsequent analysis, and are listed in Table 2 along with coupling (*n*) estimates derived from the *full calibration* and *ratio* methods, and are shown in Fig. 4.

As *n* is a ratio it is prone to extreme outliers. This is a consequence of exacerbation of errors with small (close to zero) %ΔCMRO_2_ leading to implausibly large coupling values. With *n* values derived from *full calibration* %ΔCMRO_2_ estimates, we used a conservative threshold of 5 median absolute deviations (Leys et al., 2013) from the median to iteratively remove outliers for each contrast level, which resulted in rejection of 5 out of a total of 40 data points (2 in the 1% condition, 1 in each of the others). The removal of outliers resulted in missing data points in the *full calibration* estimates of *n*. Thus, to test for significant effects of contrast and baseline on *n* using an ANOVA model, we used the *ratio* method estimates of *n*.

The *ratio* method requires an assumption for the value of *n* in the 100% contrast (see Methods section). We used an assumed value of 4.1 based on the ratio of the group average %ΔCBF and *full calibration* estimate of %ΔCMRO_2_ (*n* = CBF/ΔCMRO_2_, 4.1 = 38.2/9.36). The *ratio* method estimates of %ΔCMRO_2_ and *n* were not significantly different from the *full calibration* estimates, but were less noisy. A one-way repeated measures ANOVA using the *ratio* estimated *n* values revealed a significant effect of baseline and contrast on *n* (p < 0.01), but no interaction effect. Post-hoc paired t-tests reveal that *n* for the 1% contrast is significantly different from n for the 100% contrast for both NC (p < 0.01) and HC (p < 0.01), but not *n* for the 5 and 10% contrasts.

## Discussion

### CBF/CMRO_2_ coupling

Recent evidence suggests that the balance between evoked changes in CBF and CMRO_2_ is highly context dependent, and so is not only of interest with regard to the origin of the BOLD response, but also as a phenomenon in itself (Buxton et al., 2014). Several studies have found significant within subject variability of *n* (Ances et al., 2008, Chiarelli et al., 2007, Liang et al., 2013, Moradi et al., 2012, Moradi and Buxton, 2013, Schmithorst et al., 2015), signifying that aspects of neurovascular coupling may be modulated by properties of the stimulus or current mental state. Using a graded visual stimulus allowed us to characterise the dynamic behaviour of *n* in response to increasing stimulus intensity. In this regard, we have replicated the findings of Liang et al. (2013), by showing that *n* increases with the contrast of an 8 Hz reversing checkerboard stimulus, although unlike their study no assumptions were made about the exact amount of coupling. Using the *full calibration* method we found a much larger increase of *n* from the 1% to 100% contrast of ~ 1.5 to ~ 4, compared with ~ 1.7 to ~ 2.3 assumed by Liang et al.

The idea that there may be significant variability of *n* in the brain is of great interest as a potential insight into the mechanisms that drive CBF and CMRO_2_, and how they function in health and disease. Griffeth et al. recently hypothesized that evoked CBF responses may be more sensitive to glutamate mediated excitatory activity than CMRO_2_ changes, which more likely reflect overall energy requirements (Griffeth et al., 2015). However, they found no difference in *n* between a simple visual stimulus and a more complex ‘naturalistic’ movie stimulus, which due to feedback from higher order cognitive process would be expected to have altered the balance of excitatory activity in primary visual cortex. Thus, the mechanisms that drive these observations of within subject variability in *n* are not yet apparent, but they strongly reinforce the complex qualitative nature of the BOLD response. Therefore a better understanding of the processes that drive CBF and CMRO_2_ is required, as it could lead to further insights into the relationship between cerebral physiology and neural activity, and how that is reflected in BOLD fMRI.

Many studies have investigated %ΔBOLD and %ΔCBF dependence on CBF_0_, but with relatively little discussion on how any such dependence would translate to changes in *n*, and whether that would reflect a baseline dependence for neurovascular coupling. A calibrated BOLD study into the effects of indomethacin on BOLD responses in motor cortex, reported reduced fractional CBF responses and unaltered fractional CMRO_2_ responses, thus implying a reduction in *n* brought about by changes to baseline CBF (St Lawrence et al., 2003). However, this interpretation was challenged and it was suggested that a change in baseline CMRO_2_ with indomethacin not considered by the authors, would suggest CBF changes approximately twice as large as CMRO_2_ changes with and without the drug, implying preservation of neurovascular coupling for different baseline states (Uludag and Buxton, 2004). The primary finding of our study is that absolute ΔCBF to a graded visual stimulus was the same at two different baseline levels. Since baseline blood flow values change, the relative %ΔCBF was reduced at the higher CBF_0_. Thus, if we assume %ΔCMRO_2_ remains the same between conditions, *n* is reduced at elevated CBF_0_.

As subjects were exposed to the same visual stimuli during NC and HC periods, it seems reasonable to assume that ΔCMRO_2_ is the same for both conditions. Also assuming iso-metabolism during hypercapnia, a key principle of the calibrated BOLD methodology, %ΔCMRO_2_ remains unchanged. This necessarily implies that the coupling n is lower in the HC condition. Consistent with the elevated CBF_0_ causing changes in %ΔCBF between conditions, we saw no effect of an interaction between baseline and contrast on %ΔCBF, and therefore no interaction effect on *n*. Although values of *n* are lower at HC as a result increased CBF_0_ with preserved ΔCBF, given that the change in *n* with increased visual contrast is still present during HC, this tends to preclude an explanation based on a “ceiling” effect of CBF which would lead a reduced dynamic range of *n* during HC. Even higher levels of CBF_0_ may be required before changes in *n* become constrained by cerebrovascular reserve capacity, but our data suggest that subjects were not elevated into a flow limited regime.

### BOLD and CBF responses

We found no change in %ΔBOLD at HC, in agreement with studies that have shown steady state BOLD responses that are independent of global flow (Corfield et al., 2001, Hoge et al., 1999, Uludag et al., 2004), but contradicted by others that suggest an inverse relationship exists (Bandettini and Wong, 1997, Cohen et al., 2002, Stefanovic et al., 2006). Different groups have used different measurement techniques and methods for modulating CBF_0_, so their respective conclusions may not be generalizable to all scenarios. However, there are studies that measured BOLD responses across a range of CBF_0_, both reduced and increased from baseline via hyperventilation and hypercapnia respectively (Kemna and Posse, 2001, Posse et al., 2001, Weckesser et al., 1999). Results from these investigations indicate a nonlinear relationship whereby BOLD contrast is reduced at both extremes of high and low CBF_0_. In this study, the HC challenge increased P_ET_CO_2_ from a mean (± SD) of 37.0 ± 2.46 to 43.6 ± 2.64, which falls within the linear range reported by Posse et al. (2001), and so the slight non-significant increase in %ΔBOLD that we observe is consistent with these findings. It has also been suggested that the discrepancy in the literature might be explained by the experimental design and whether or not a steady state of hypercapnia has been reached (Liu et al., 2007), with those studies like ours reporting BOLD responses that are independent of flow, likely to still be in the transient state (Liu et al., 2007). We allowed a 60 s transition period and manually targeted P_ET_CO_2_ levels with CO_2_ pre-emphasis, which in theory should allow us to reach a steady state more quickly than the estimated 120–150 s that it takes if P_ET_CO_2_ are allowed to naturally fluctuate in response to a fixed inspired hypercapnia challenge (Liu et al., 2007).

The few studies that have investigated functional CBF responses at different CBF_0_ provide an unclear picture. Some have found no relationship between global and absolute CBF changes during neural stimulation (Kastrup et al., 1999, Li et al., 2000), and thus an inverse relationship with fractional changes, whereas Shimosegawa et al. found that ΔCBF changes were proportional to CBF_0_, meaning that %ΔCBF changes remained constant (Shimosegawa et al., 1995). Stefanovic et al. used a range of fixed inspired hypercapnia challenges (5, 7.5, and 10% CO_2_), and reported an inverse relationship between focal CBF activations and global flow with linear fit slopes of − 0.18 ± 0.02%/% and − 0.13 ± 0.01%/% in the motor and visual cortices respectively (Stefanovic et al., 2006). Many of the inconsistencies in the literature may be related to different levels of CO_2_ used, making meaningful comparisons between studies difficult. It is particularly problematic when larger concentrations of CO_2_ (> 5%) are used, as that is likely to introduce additional confounds related to subject tolerance and sensitivity to hypercapnia, and possible resting CMRO_2_ changes (Driver et al., 2015), all of which will contribute to estimates of focal activations of BOLD and CBF. Therefore, further study into the relationship between global flow and evoked BOLD and CBF responses, which carefully control for these potential serious confounds, is required.

### Limitations of the study

A limitation of the study is that we were unable to obtain accurate individual subject %ΔCMRO_2_ for the HC condition. We have assumed that absolute ΔCMRO_2_ will be the same in NC and HC as the “amount” of neural activity, and thus energy requirement, should be equal. This is supported by non-human studies showing stimulus evoked CMRO_2_ changes correspond well with local neuronal firing rates (Kida and Hyder, 2006, Maandag et al., 2007, Smith et al., 2002). Human electrophysiology studies that have specifically looked at the effect of hypercapnia are less clear, with one magnetoencephalography (MEG) study reporting motor and visual stimulus induced fractional changes in neuronal oscillations that were unaffected by hypercapnia (Hall et al., 2011), and another finding that event-related fields were reduced (Thesen et al., 2012). However, these MEG studies do not directly measure CMRO_2_. Therefore, the assumption that %ΔCMRO_2_ is the same at HC relies on there being no CO_2_ induced change in baseline CMRO_2_, which is a contentious issue. MRI studies tend to show no change or small changes (< 15%) in CMRO_2_ (Chen and Pike, 2010, Jain et al., 2011, Xu et al., 2011, Zappe et al., 2008), and more direct measures of neural activity obtained with electroencephalography (EEG) and MEG data show broadband reductions in oscillatory amplitude (Driver et al., 2015, Hall et al., 2011, Xu et al., 2011).

**Fig. 5:**
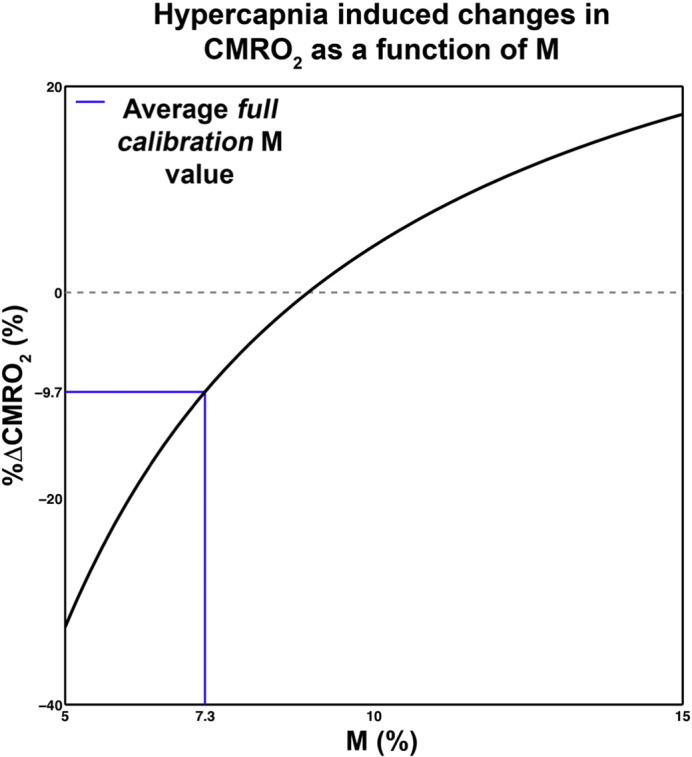
Approximate group level %ΔCMRO_2_ in response to the hypercapnia challenge as a function of M. The group mean full calibration M corresponds with %ΔCMRO_2_ to − 9.7%.

As we have the same stimulus during NC and HC, using group-averaged %ΔBOLD and %ΔCBF from the NC baseline, we can formulate a rough estimate of the %ΔCMRO_2_ due to HC (%ΔCMRO_2|HC_) with the Davis model as follows. The Davis model for evoked visual fractional BOLD responses in the NC (Δ*S*_*v*_/*S*_0_) and HC ($\Delta S_{v+CO_{2}}/S_{0}$) conditions are given as:

(2) $$\frac{\Delta S_{V}}{S_{0}}=M\left[1-{\left(1+\frac{\Delta CBF_{v}}{CBF_{0}}\right)}^{\alpha -\beta }{\left(1+\frac{\Delta CMRO_{2|v}}{CMRO_{2|0}}\right)}^{\beta }\right]\text{.}$$

(3) $$\frac{\Delta S_{V+CO_{2}}}{S_{0}}=M\left[1-{\left(1+\frac{\Delta CBF_{v+CO_{2}}}{CBF_{0}}\right)}^{\alpha -\beta }{\left(1+\frac{{\mathrm{\Delta CMRO}}_{2|v}}{CMRO_{2|0}}+\frac{\Delta CMRO_{2|CO_{2}}}{CMRO_{2|0}}\right)}^{\beta }\right]$$

where the subscripts v and CO_2_ denote visual and hypercapnia induced changes respectively. Rearranging the equations and providing an estimate of M, %ΔCMRO_2|HC_ is simply the difference between CMRO_2_ terms in the BOLD model for the NC and HC conditions. Using group average mean %ΔBOLD and %ΔCBF for all contrasts, we can approximate %ΔCMRO_2|HC_. Fig. 5 shows the approximated %ΔCMRO_2_|_HC_ as a function of the maximum BOLD change parameter M.

For the *full calibration* average M value that we obtained, the data we have supports an approximate ~ 10% reduction in CMRO_2_, which is in good agreement with Xu et al. who directly addressed this issue, and reported a CMRO_2_ reduction of ~ 13% during hypercapnia (Xu et al., 2011). If %ΔCMRO_2_|_HC_ is indeed negative, given that our *full calibration* M estimates assume iso-metabolism, they are over estimated, in which case the approximation of 10% is an upper limit on the reduction in CMRO_2_ associated with the HC challenge. Visual stimulus evoked %ΔCMRO_2_ will therefore be very similar for NC and HC, and will have a limited impact on the HC reduced *n* values compared with the large (~ 40%) differences in %ΔCBF. Given the reduced %ΔCBF at HC, for visual stimulus *n* values to be the same as NC, %ΔCMRO_2_ would have to be significantly smaller. This would require either an increase in baseline CMRO_2_ due to hypercapnia (with unchanged ΔCMRO_2_), or a decrease in stimulus evoked ΔCMRO_2_, which is unlikely. Simply stated, the assumption that %ΔCMRO_2_ does not change in response to the hypercapnia challenge, does not critically sway our interpretation of the data.

It has previously been shown that caffeine, which has a large effect on baseline CBF, can alter the spatial extent of CBF activations (Liau et al., 2008). If the spatial extent differs between NC and HC, a common ROI for both baseline states could be biased towards one particular condition. To rule out this possibility, we also looked at BOLD and CBF activations from separate ROIs that were created as above (see Methods section), but separately for the NC and HC conditions. The number of visual stimulus activated voxels (mean ± SD) was 207 ± 94 and 216 ± 127 in the NC and HC conditions respectively, with no significant difference. Repeated measures ANOVAs were constructed to look specifically at the effect of ROI choice on responses. The separate NC and HC ROIs did yield larger %ΔBOLD, %ΔCBF, and ΔCBF activations than the common ROI (p < 0.01) as expected due to fewer “noisy” voxels, but there were no significant interactions with contrast or baseline. This suggests that baseline effect of %ΔCBF is not driven by the differences in activated voxels in the two conditions.

The Davis model is a steady state description of the BOLD response, a process that in reality is dynamic, with the balance of different contrast sources changing during its evolution. In particular the model assumes that CBV changes are largely a passive product of CBF changes, and thus the two quantities can be linked through an invariant coupling parameter α, however evidence from animal studies suggests a slowly evolving venous CBV component exists that becomes detectable after approximately 30 s of stimulation (Drew et al., 2011, Kim and Kim, 2011, Zong et al., 2012). Although our choice of a 30 s stimulus duration should limit this potential source of error, it should be noted that our experimental design includes periods where different contrast levels are presented in succession without separation by a baseline period. Thus, we need to be wary of the effect of slow transient venous CBF changes across back-to-back 30 s stimulus presentations. It is not immediately obvious how this might impact our CMRO_2_ estimates, particularly given that our choice of an optimised value of α no longer carries its original physiological meaning (Griffeth and Buxton, 2011), and given that CBF “state” changes from one 30 s presentation to the next. The pseudorandom presentation order would be expected to lessen any hypothetical effect, and our finding of *n* changing with contrast level is a replication of Liang et al, who used 20 s visual stimuli that were always interleaved with 40 s of baseline (Liang et al., 2013). This would suggest that our experimental design does not introduce a significant source of error from slow venous CBV changes. Even so, this is still an important area of investigation for future studies, especially in leading towards a more dynamic description of neurovascular coupling.

## Conclusions

Further work to characterise the dynamic range of *n* for a graded stimulus across multiple baseline perfusion levels is needed, as it could potentially provide a marker of cerebrovascular health. Here we show that ΔCBF is independent of CBF_0_ and therefore conceivably more representative of neurovascular coupling than %ΔCBF. This finding has implications for previous studies that have measured regional variations in *n* (Ances et al., 2008, Chiarelli et al., 2007), given that baseline CBF has been shown to vary across different cortical areas (Murphy et al., 2011). Additionally, these data are an extension of the original discovery that *n* is dependent on stimulus intensity (Liang et al., 2013); in that they demonstrate that this effect is conserved at a different baseline level of perfusion, suggesting that it is a fundamental feature of the brain's neurovascular response to the stimuli. Newly developed calibration methods combining hypercapnia and hyperoxia allow oxygen extraction fraction (OEF) and absolute CMRO_2_ to be estimated (Bulte et al., 2012, Gauthier and Hoge, 2013, Wise et al., 2013), and will be important in fully characterising the metabolic and vascular responses to neural activity, and separating out the effects of baseline.

These results highlight the need to better understand the role of CBF/CMRO_2_ coupling in functional activations in the human brain. Absolute CBF changes during NC and HC more accurately predict relative BOLD response in the two different baseline states than fractional CBF changes. Thus, the BOLD response may be more sensitive to a measure of neurovascular coupling based on absolute CBF changes (e.g. *n*_*abs*_ = Δ*CBF*/Δ*CMRO*_2_). Such a measure, with physiologically meaningful units (mL/μmol), would provide a quantitative gauge of the brain's hyperaemic response to a given metabolic demand, which is closely matched to underlying neural activity. Further studies comparing the absolute and fractional CBF and CMRO_2_ responses at different baselines will be important for elucidating the most informative measure of neurovascular coupling with regard to brain function and the BOLD response.

## Figures and Tables

**Table 1:** List of variables used and corresponding units.

Variable	Description	Units
S_0_	Baseline BOLD signal	Arbitrary units
ΔS	Change in BOLD signal from baseline S_0_	Arbitrary units
%ΔBOLD	Fractional change in BOLD signal ($\frac{\Delta S}{S_{0}}$)	Fraction (%)
CBF_0_	Baseline CBF	mL 100 g^− 1^ min^− 1^
ΔCBF	Change in CBF from baseline	mL 100 g^− 1^ min^− 1^
%ΔCBF	Fractional change in CBF ($\frac{\Delta CBF}{CBF_{0}}$)	Fraction (%)
CMRO_2|0_	Baseline CMRO_2_	μmol 100 g^− 1^ min^− 1^
ΔCMRO_2_	Change in CMRO_2_ from baseline	μmol 100 g^− 1^ min^− 1^
%ΔCMRO_2_	Fractional change in CMRO_2_ ($\frac{\Delta CMRO_{2}}{CMRO_{2|0}}$)	Fraction (%)

**Table 2:** Measured group average responses (± SD) for each contrast level.

BOLD and CBF responses						
Contrast (%)	%ΔBOLD (%)		%ΔCBF (%)		ΔCBF (mL/100 g min)	
NC	HC	NC	HC	NC	HC
1	0.26 ± 0.26	0.28 ± 0.17	11.5 ± 5.28	6.52 ± 3.19	6.00 ± 3.07	5.00 ± 2.30
5	0.55 ± 0.28	0.67 ± 0.15	19.6 ± 5.40	12.5 ± 4.56	10.3 ± 3.55	9.74 ± 3.93
10	0.74 ± 0.20	0.73 ± 0.13	25.6 ± 7.27	15.4 ± 5.55	13.0 ± 3.48	11.7 ± 4.09
100	1.09 ± 0.28	1.11 ± 0.21	38.2 ± 7.13	26.1 ± 8.96	19.7 ± 5.00	20.1 ± 6.41


%CMRO_2_ responses and CBF/CRMO_2_ coupling (*n*)						
Contrast (%)	%ΔCMRO_2_ (%)		*n* full calibration		*n* ratio method	
NC		NC	HC	NC	HC

1	6.90 ± 4.62		1.54 ± 0.79	0.87 ± 0.52	1.96 ± 1.31	1.09 ± 0.68
5	7.04 ± 4.81		3.36 ± 2.13	2.12 ± 1.36	3.16 ± 1.64	2.21 ± 1.49
10	8.37 ± 5.69		3.63 ± 1.94	2.16 ± 1.41	3.48 ± 1.45	2.13 ± 1.04
100	9.36 ± 3.72		4.03 ± 1.29	2.56 ± 0.77	4.1 (assumed)	2.89 ± 1.16
